# Effects of high-intensity interval training and moderate-intensity continuous training on glycaemic control and skeletal muscle mitochondrial function in db/db mice

**DOI:** 10.1038/s41598-017-00276-8

**Published:** 2017-03-16

**Authors:** Vivien Chavanelle, Nathalie Boisseau, Yolanda F Otero, Lydie Combaret, Dominique Dardevet, Christophe Montaurier, Geoffrey Delcros, Sébastien L Peltier, Pascal Sirvent

**Affiliations:** 1 Université Clermont Auvergne, Laboratoire des Adaptations Métaboliques à l’Exercice en conditions Physiologiques et Pathologiques (AME2P), Clermont-Ferrand, F-63000 France; 2 Valbiotis S.A.S., La Rochelle, France; 3 INRA, Unité de Nutrition Humaine (UNH, UMR 1019), CRNH Auvergne, France

## Abstract

Physical activity is known as an effective strategy for prevention and treatment of Type 2 Diabetes. The aim of this work was to compare the effects of a traditional Moderate Intensity Continuous Training (MICT) with a High Intensity Interval Training (HIIT) on glucose metabolism and mitochondrial function in diabetic mice. Diabetic db/db male mice (N = 25) aged 6 weeks were subdivided into MICT, HIIT or control (CON) group. Animals in the training groups ran on a treadmill 5 days/week during 10 weeks. MICT group ran for 80 min (0° slope) at 50–60% of maximal speed (Vmax) reached during an incremental test. HIIT group ran thirteen times 4 minutes (20° slope) at 85–90% of Vmax separated by 2-min-rest periods. HIIT lowered fasting glycaemia and HbA1c compared with CON group (p < 0.05). In all mitochondrial function markers assessed, no differences were noted between the three groups except for total amount of electron transport chain proteins, slightly increased in the HIIT group vs CON. Western blot analysis revealed a significant increase of muscle Glut4 content (about 2 fold) and higher insulin-stimulated Akt phosphorylation ratios in HIIT group. HIIT seems to improve glucose metabolism more efficiently than MICT in diabetic mice by mechanisms independent of mitochondrial adaptations.

## Introduction

Overall, physical activity appears as an effective strategy in both prevention and treatment of Type 2 Diabetes (T2D)^1^. Numerous studies have shown improved insulin sensitivity, glucose tolerance and long term glucose control following exercise training interventions^2–4^, and current guidelines from the major international scientific organizations regarding prescriptions of aerobic training in T2D now recommend 150 min of moderate to vigorous intensity exercise weekly^5^. Nevertheless, long-term adherence to physical activity in individuals with T2D is problematic^6^. Moreover, some studies have reported resistance to the beneficial effects of exercise in some individuals^7^. Therefore, identification of physical activity modalities displaying higher adherence and/or efficiency is a major challenge to improve health benefits in individuals with T2D.

It has been demonstrated that high-intensity interval training (HIIT) can improve patient adherence to physical activity^8, 9^, and numerous recent studies have also shown improvements in glycaemic control following HIIT^10–19^. The effects of HIIT on blood glucose could be partly mediated by improved skeletal muscle mitochondrial function, as it has been suggested by some authors^20^. HIIT seems indeed to be a strong activator of mitochondrial biogenesis and this effect could be mediated by stimulation of PGC1-α expression^21–24^. Mitochondrial impairment has been linked to insulin resistance^25^, and consequently, improvement of mitochondrial function may restore skeletal muscle insulin signalling^26^. To date, no study has compared the effects of HIIT *vs*. the traditional moderate-intensity continuous training (MICT) on skeletal muscle mitochondrial adaptations in a context of T2D.

Consequently, the aim of this work was to compare the effects of 10 weeks of HIIT and MICT on glycaemic profile, skeletal muscle insulin signalling and mitochondrial function in diabetic mice. We hypothesised that HIIT would lead to greater benefits on glycaemic control due to superior mitochondrial function improvements.

## Results

### Body composition and indirect calorimetry measurements

No significant effects of training were noted on body weight or fat and lean mass compared with CON (Table 1).

**Table 1:** Mice characteristics before and after training.

	*CON*	*MICT*	*HIIT*	*p* (*ANOVA*)
N=	9	8	8	
Body mass week 10 (g)	39.89 ± 0.79	39.04 ± 1.43	35.19 ± 3.19	0.232
Fat mass week 1 (% b.w.)	47.46 ± 0.76	46.48 ± 0.74	48.96 ± 0.50	0.060
Fat mass week 10 (% b.w.)	52.96 ± 0.78	53.11 ± 0.75	48.14 ± 4.31	0.920
Lean mass week 1 (% b.w.)	49.66 ± 0.74	50.81 ± 0.97	48.67 ± 0.54	0.250
Lean mass week 10 (% b.w.)	44.91 ± 0.62	44.95 ± 0.66	50.27 ± 4.18	0.710
Liver mass (g)	1.70 ± 0.09	1.68 ± 0.07	1.48 ± 0.07	0.093
Liver mass (% b.w.)	4.27 ± 0.19	4.35 ± 0.25	4.54 ± 0.06	0.875
Gastroc mass (mg)	51.75 ± 2.80	51.50 ± 3.58	49.87 ± 4.28	0.920
Gastroc mass (% b.w.)	0.13 ± 0.01	0.13 ± 0.01	0.15 ± 0.01	0.372
Liver TG (nmol/mg)	47.86 ± 1.68	47.65 ± 5.34	36.31 ± 4.93	0.118

### Glycaemia and response to starch and insulin tolerance tests

**Figure 1:**
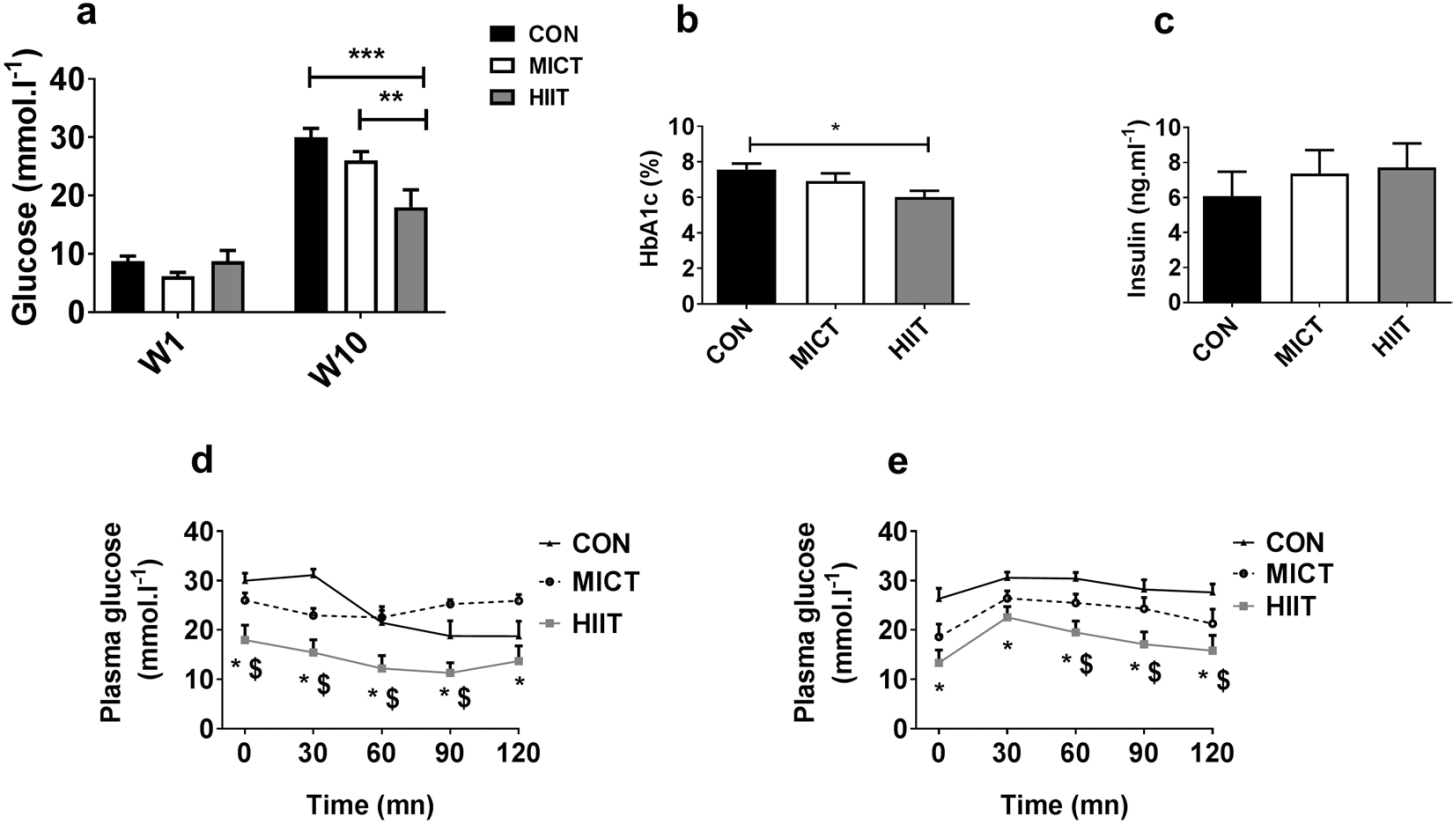
Effects of 10 weeks of MICT or HIIT on fasting blood glucose (**a**), glycosylated haemoglobin (**b**), fasting serum insulin (**c**) and blood glucose response to ITT (**d**,**f**) and OSTT (**e**,**g**). Data are expressed as means ± SEM. *p < 0.05; **p < 0.01; *Significantly different from CON; ^$^Significantly different from MICT.

After 10 weeks of training, fasting plasma glucose was significantly lower in HIIT group (18.0 ± 3.0 mmol.l^−1^) than in MICT group (26.0 ± 1.5 mmol.l^−1^, p < 0.05) and CON group (30.0 ± 1.5 mmol.l^−1^, p < 0.01) (Fig. 1a). In accordance with this result, HbA_1c_ level was also significantly reduced in HIIT group (6.0 ± 0.4%) compared with CON group (7.5 ± 0.3%, p < 0.05) (Fig. 1b). Fasting serum insulin levels were not different among the groups (Fig. 1c). Repeated measures ANOVA of ITT (Fig. 1d) and OSTT (Fig. 1e) revealed significant differences between groups at several time points. However, since AUC_net_ (baseline-corrected) did not differ between groups (not shown), it is concluded that these differences merely reflect differences in fasting glucose levels.

### Muscle oxidative metabolism

**Figure 2:**
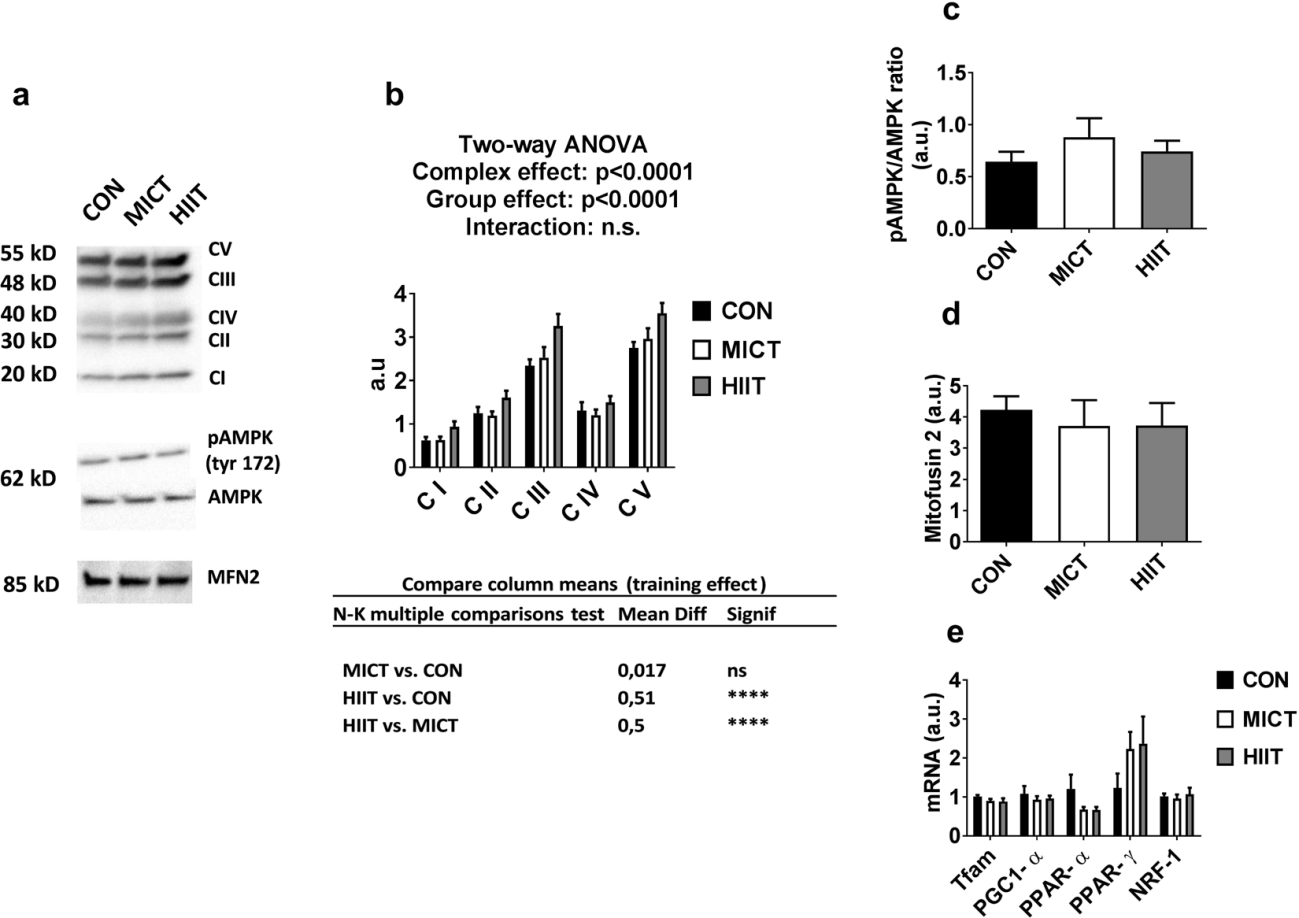
(**a**) Western blot representative images. The blots were cropped and full length blots are presented in Supplementary Figure S1. (**b**) ETC protein content assessed by western blot. (**c**) p-AMPKα Thr172/total-AMPK ratio. (**d**) Mitofusin levels assessed by western blot. (**e**) mRNA expression of mitochondrial biogenesis marker genes. Data are expressed as means ± SEM. ****p < 0.0001.

**Figure 3:**
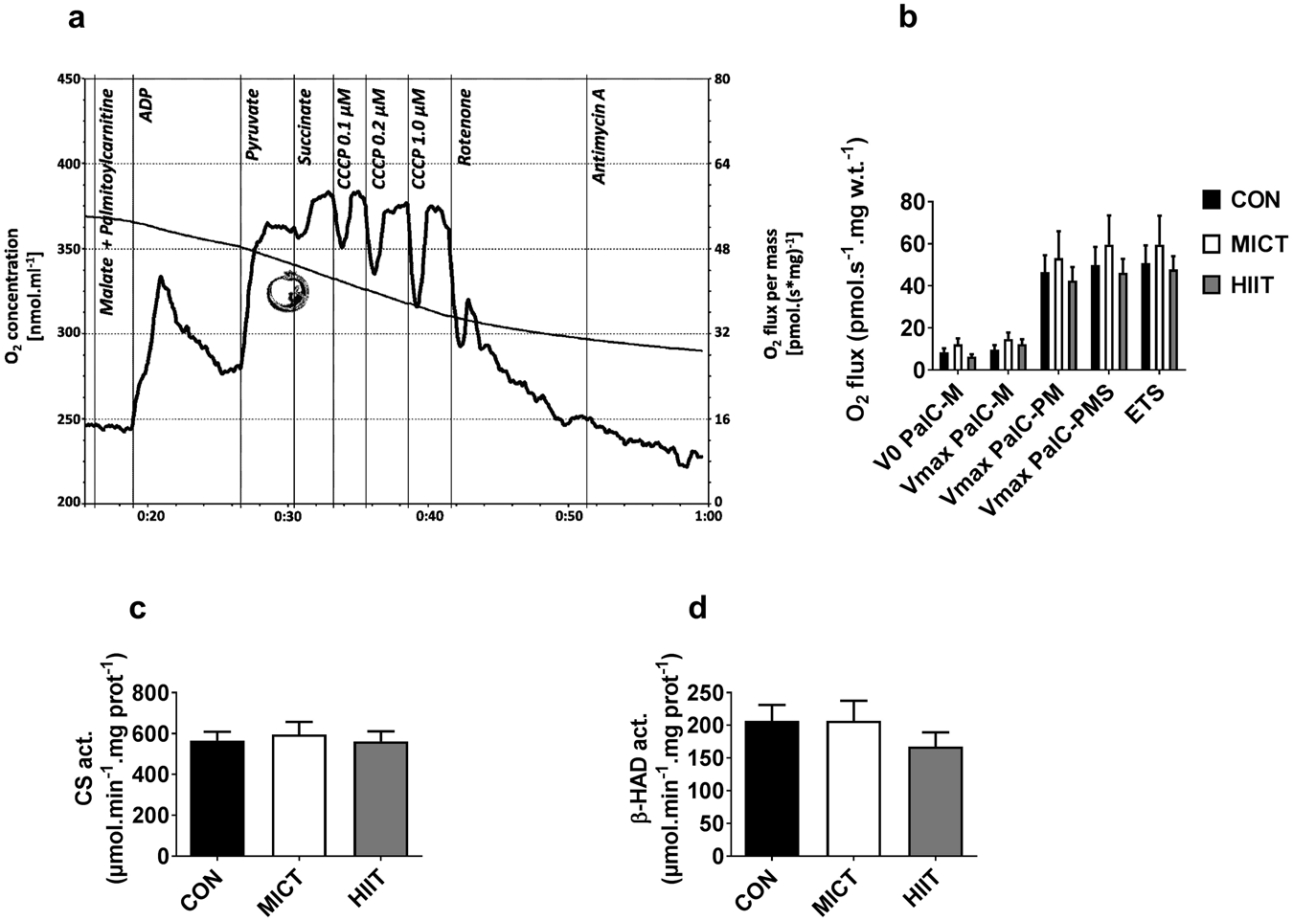
Oxygen concentration and mass-specific oxygen flux as a function of time were recorded in a SUIT protocol with permeablised gastrocnemius. 0.02 mM palmitoyl-carnitine (Pal-C) and 2 mM malate were added in the chambers before the fibres were added. (ADP: 2.5 mM adenosine diphosphate; P: 5 mM pyruvate; S: 10 mM succinate; U: Carbonyl cyanide m-chloro phenyl hydrazine (CCCP); Rot: 0.5 µM; AmA: 2.5 µM Antimycin A). (**a**) Representative measurement of mitochondrial respiration. (**b**) Effects of MICT and HITT on mitochondrial respiration. (**c**) Citrate synthase and (**d**) β-hydroxyacyl-CoA dehydrogenase activities. Data are expressed as means ± SEM.

In *gastrocnemius*, western blot analysis of OXPHOS ETC revealed a modest but significant global increase of the total amount of ETC proteins for HIIT group vs MICT and CON groups (p < 0.0001, Fig. 2b). AMPK is known as an important mediator of mitochondrial adaptations to exercise training, mainly through regulation of PGC1-α^27^. We found here that p-AMPK Thr172/AMPK ratio was unaffected by exercise (Fig. 2c). OXPHOS ETC, AMPK and p-AMPK full-length blots are presented in Supplementary Figure S1. In accordance, no effects of exercise training were seen on mRNA levels of PGC1-α, or other known regulators of mitochondrial biogenesis (Tfam, PPAR-α, PPAR-γ, NRF-1; Fig. 2e). Similarly, protein levels of mitofusin-2 (MFN2), a marker of mitochondrial fusion that has been shown to be repressed in diabetic state and increased following exercise^28^, were not modified by MICT or HIIT protocols (Fig. 2d). MFN2 full-length blots are presented in Supplementary Figure S1. In line with these results regarding mRNA and protein levels of known regulators of mitochondrial function, we did not find any modification in CS and β-HAD activities caused by exercise training (Fig. 3c and d). We also measured leak state (V_0_) and maximal respiration (V_max_) of *gastrocnemius* permeabilised fibres during a SUIT protocol corrected for ROX and instrumental background. No differences were seen between groups regarding maximum steady state O_2_ flux after sequential addition of palmitoylcarnitine and malate (V_0_ PalC-M), ADP (V_max_ PalC-M), pyruvate (V_max_ PalC-PM), succinate (V_max_ PalC-PMS) and CCCP (ETS) (Fig. 3b).

### Insulin signalling and Glut4 levels in skeletal muscle

**Figure 4:**
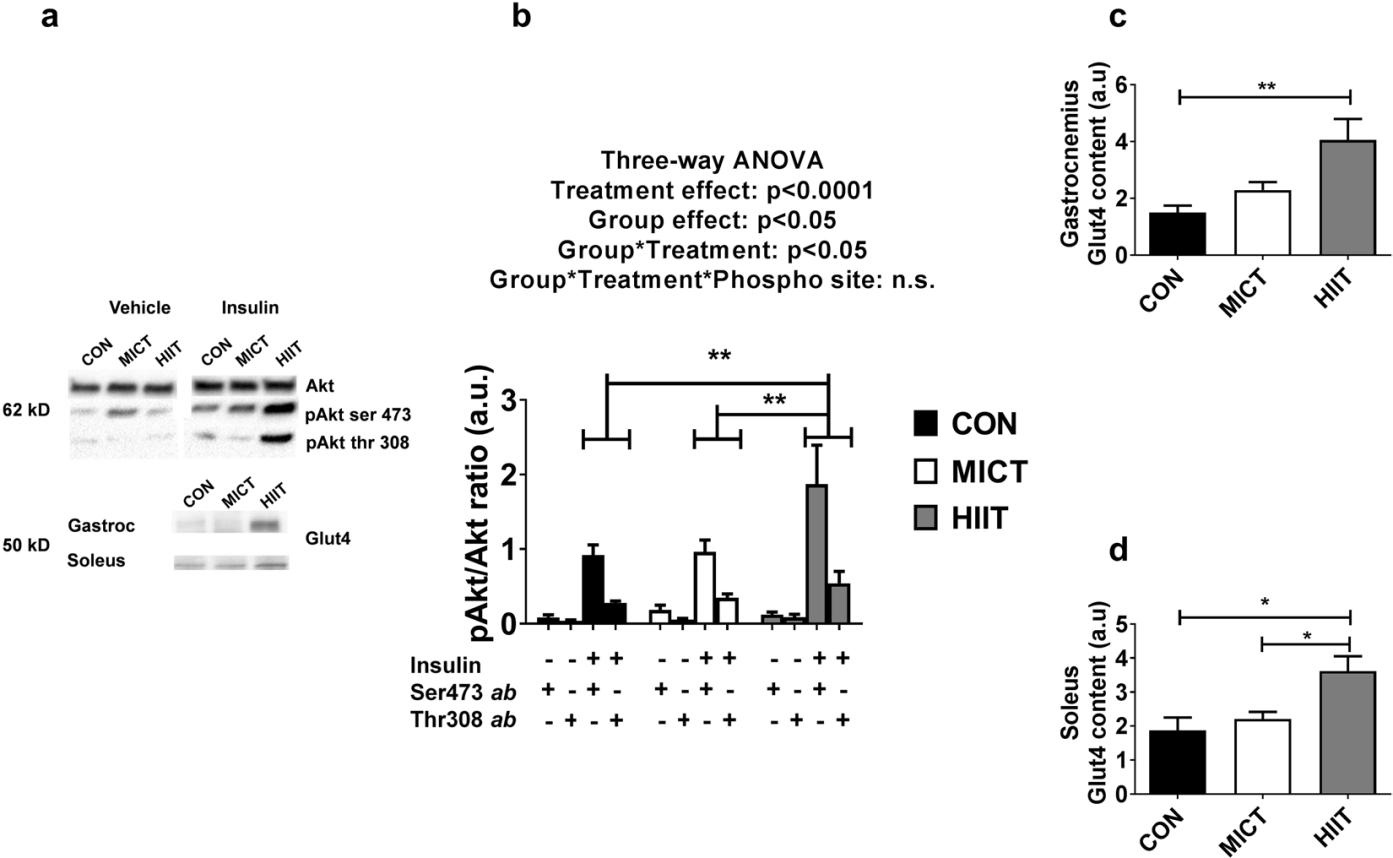
(**a**) Western Blot representative images. The blots were cropped and full length blots are presented in Supplementary Figure S2. (**b**) In soleus, phosphorylation ratios of insulin-stimulated Akt ser473/total-Akt and Akt thr 308/total-Akt. Gastrocnemius (**c**) and soleus (**d**) Glut4 content assessed by western blot. Data are expressed as means ± SEM. *p < 0.05; **p < 0.01.

*Ex vivo* basal and insulin-stimulated phosphorylation of Akt on Ser473 and Thr308 were investigated in paired *soleus* muscles incubated in presence or absence of 12 nM insulin. ANOVA showed a significant effect of exercise training (p < 0.05), and a significant interaction (exercise*insulin, p < 0.05). Post-hoc analysis revealed a higher insulin-stimulated Akt phosphorylation state in HIIT compared with MICT (p < 0.05) and CON (p < 0.05) (Fig. 4a). Glut4 content was significantly higher in HIIT group when compared with CON in *gastrocnemius* (about 2.5 fold, p < 0.01) and *soleus* (about 2 fold, p < 0.05) (Fig. 4b and c). *Soleus* Glut4 content was also increased in HIIT compared with MICT group (Fig. 4c, p < 0.05). All full-length blots are presented in Supplementary Figure S2.

## Discussion

The results of the present study lead to confirm the hypothesis that HIIT improves glucose metabolism in a superior manner than MICT. We did not observe major changes in the mitochondria in any of the trained groups, suggesting that mitochondrial adaptations were not involved in such effect. Skeletal muscle insulin signalling and Glut4 content were only improved in the HIIT group, suggesting that skeletal muscle insulin sensitivity would be more altered by the effects of HIIT than by those of MICT. This could account for the better improvement of glucose homeostasis following HIIT intervention.

We chose in this work to compare the effects of two exercise training modalities in db/db mice. These animals quickly exhibit massive obesity and develop diabetes because of a mutation in the leptin receptor gene^29^. In fact, there is no “perfect” model to study T2D. This pathology is very complex, because of the multiplicity of its genetic and environmental determinants^30^. Therefore, although this db/db monogenic model of diabetes is naturally not representative of all forms of the disease, this animal model is among the most often utilised and widely accepted for treatment testing in diabetes research^31^. Leptin resistance is a common feature of obesity-related T2D^32^, and db/db mice represent an interesting model sharing most of metabolic abnormalities observed in human subjects^31^. Moreover, it has already been demonstrated that mitochondrial adaptations to exercise may be inhibited in the absence of leptin^33^. In this context, one could hypothesize that mitochondrial adaptations to exercise in the skeletal muscle might be difficult to obtain in db/db mice. Similarly, it has been shown that diabetic patients qualified of “exercise resistant” (*e*.*g*. failing to improve HbA1c in response to an exercise training program) display a concomitant incapacity to stimulate mitochondrial biogenesis^7, 34^. The db/db mouse therefore appears as a good model to study the efficiency of exercise training in a “difficult” context, well representative of “exercise resistant” diabetic patients. However, it would still be interesting to reproduce this protocol in different T2D models without monogenic cause.

We observed lower fasting glycaemia confirmed by lower HbA1c in HIIT group despite the absence of effect on body composition, whereas no improvements were noted in MICT group. To our knowledge, this study was the first to compare HIIT vs MICT in a context of T2D. An overview of the present literature testifies to the difficulty to obtain beneficial changes induced by physical activity in db/db mice. Two research teams have tested HIIT in this model with a similar protocol. Ostler *et al*. showed modest improvements in glucose tolerance and basal Akt Ser473 phosphorylation^35^. Stølen *et al*. noted an improvement in lipid profile^36^. However, none of these teams showed lower fasting glucose. Among authors having tested more traditional continuous endurance exercise on db/db mice, Lee *et al*. showed improvements on insulin tolerance^37, 38^ with no changes on fasting glucose, using a comparable MICT protocol as ours. Other studies on this model have failed to improve glycaemic control with such a protocol^39–41^. Therefore, our study confirms that exercise-training adaptations regarding glucose homeostasis are difficult to obtain in db/db mice, reinforcing the idea that these animals could be used as an “exercise resistance” model. Interestingly, positive effects of HIIT on glucose homeostasis were found in this context. These results strengthen the interest to study the effects of HIIT in diabetic context and highlight this exercise modality as a candidate to overpass the exercise-resistant state.

Few changes occurred following 10 weeks of HIIT in skeletal muscle mitochondrial parameters. We solely noted an increase in the total amount of ETC proteins, with no incidence on functional oxidative capacity assessed through *ex vivo* permeabilised fibres respiration. Accordingly, CS and β-HAD activities, like mRNA expression of genes related to mitochondrial biogenesis, were also left unaffected by HIIT or MICT. In wild-type mice, chronic exercise is a well-known activator of AMPK, which in turn triggers mitochondrial biogenic response through PGC1-α induction^33^. This mechanism has also been confirmed in humans^42^. These new data show that this mutant model of T2D mice did not respond to stimulatory effects of chronic exercise in any of these parameters, suggesting that some step of the signalling pathway might be defective. As it has already been suggested in experiments using ob/ob mice, intact leptin signalling might be necessary for exercise-induced adaptation through AMPK and PGC1-α in muscle^33, 35^, this could therefore explain the nearly absence of mitochondrial adaptation consecutive to exercise training in this work. These results obtained with db/db mice are also consistent with some observations made in humans. Indeed, it has also been demonstrated that the chronic exercise-induced rise in PGC1-α and Mfn2 is sometimes abolished in young type 2 diabetic subjects^43^. It is important to specify that all the experiments performed on skeletal muscle in the present study have been realised in tissues harvested 72 h after the last exercise session, to avoid the potential confounding effects of acute exercise^44^. Therefore, we cannot rule out the possibility that transient elevation of AMPK activation and PGC1-α induction may have occurred following each training session. Such effect could explain the slight increase in the amount of ETC proteins following HIIT. Nevertheless, the fact that mitochondrial respiration measurements did not exhibit any improvement in the trained groups rather indicates minor or absent functional adaptations of mitochondria following chronic exercise. Overall, these results do not argue the involvement of mitochondrial adaptations in the beneficial effects of HIIT on glycaemic control.

Although it is believed that mitochondrial adaptations may mediate part of the beneficial effects of physical activity in the case of T2D^20^, the present study suggests that non-mitochondrial-dependent mechanisms could also be involved to explain the improvements in glycaemic control after HIIT intervention. We found a significant increase for insulin-stimulated Akt phosphorylation at Ser473 and Thr308 in HIIT group vs CON. Interestingly, decreased Akt phosphorylation at one or the other of those sites is a hallmark of skeletal muscle insulin signalling defect in T2D^45, 46^. Therefore, an improvement in this parameter might reflect lower insulin resistance and might partly explain the positive effects of HIIT on fasting blood glucose concentration. Moreover, Glut4 content in *soleus* and *gastrocnemius* was significantly increased in HIIT group vs. CON and interestingly, Glut4 seems to be largely involved in whole body glucose homeostasis. Several studies using muscle-specific Glut4 KO mice have shown that Glut4 disruption can lead to hyperglycaemia^47^ or impaired glucose tolerance in high fat or chow-fed mice^48, 49^. Likewise, studies dealing with the effect of a single exercise session on Glut4-deficient mice, all converge upon the conclusion that Glut4 is the major factor responsible for exercise-induced glucose uptake^50–52^. Reciprocally, overexpression of Glut4 in both lean and diabetic db/db mice improved both fasted and fed glycaemia, insulin action, and enhanced disposal of an oral glucose challenge^53–56^. Interestingly, during acute exercise, mice overexpressing Glut4 seem to preferably utilise carbohydrates over lipids as a fuel source, so that there is no change in overall energy expenditure^57^. However this statement was challenged by a more recent study using 2-Deoxy-[3H]glucose, which showed no differences between wild type and transgenic mice in muscle glucose uptake during exercise^58^, suggesting that a normal Glut4 content is sufficient for increased glucose uptake in the muscle, during exercise. In another study, Ikemoto *et al*. demonstrated that chronic exercise resulted in increased Glut4 protein expression in both control and transgenic mice overexpressing Glut4. The authors showed a concomitant and cumulative improvement in glycaemic control associated with the enhanced Glut4 content, whether be from transgenic manipulation, physical training or both^59^. Therefore, in the present study, the increase in muscle Glut4 content induced by HIIT is therefore likely to be partly responsible for the improved ability for muscle to uptake blood glucose.

The explanation of the higher benefits of HIIT over MICT regarding improvement of insulin signalling and Glut4 content in skeletal muscle remains unknown. A recent study in healthy and obese mice has evaluated the impact of exercise intensity on the physiological adaptations to exercise training program^60^. This study showed that higher exercise intensity allowed better improvement of skeletal muscle Glut4 content and glycaemic control in obese animals. Furthermore, some signalling pathways seem particularly sensitive to exercise intensity. For example, Egan *et al*. have shown in human skeletal muscle that phosphorylation of HDAC, a well-known activator of Glut4 transcription^61^, was increased following a single bout of exercise at 80% of VO_2_peak but not after a single bout of exercise at 40% of VO_2_peak^62^. Further studies are needed to better understand the molecular mechanisms leading to higher benefits of HIIT over MICT regarding Glut4 and insulin signalling adaptations in db/db mice. Finally, it bears mentioning that exercise is an important stimulus for ATP turnover. In this study, although the duration of the exercise sessions was the same for MICT and HIIT, we were not technically able to assess energy expenditure during training. Thus, we cannot exclude that the higher energy output leading to higher ATP turnover during HIIT may also be a participating factor in the reduced fasting glucose in this group.

In summary, we showed that HIIT allowed a better improvement in glycaemic control than MICT in db/db mice. This effect was not associated with mitochondrial adaptations in the skeletal muscle. HIIT was the only exercise modality to improve skeletal muscle insulin signalling and Glut4 content. Overall, these experiments reinforce the interest of HIIT interventions in diabetic patients. In addition to the claim of better adherence for patients to this modality, HIIT may also exhibit higher beneficial effects on metabolic adaptations. Further studies are needed to confirm these effects and to elucidate the explanatory physiological mechanisms.

## Methods

### Animals

All methods were carried out in accordance with *the guide for the care and use of laboratory animals*, Eighth edition (2011). All procedures were approved by local ethic committee (CE2A-02, Auvergne, France). Male db/db (BKS(D)-Leprdb/JOrlRj) aged 5 weeks were obtained from Janvier (JANVIER LABS, France), housed in individual cages with normal 12-h: 12-h light/dark cycles and fed a standard diet (A03 enriched with 3% corn oil, Safe, France). After 1 week of acclimatization, 30 mice out of 46 were selected for the study after a running test and the best runners were randomly divided into control group (CON), MICT and HIIT. After 10 weeks of training, animals were fasted overnight and killed by cervical dislocation before tissues were harvested.

### Exercise performance test

The protocol was based on previous published works^35, 37, 63^ with the following adaptations: The mice from MICT group were placed on the treadmill and warmed up during 5 min at a speed of 6 m/min. Speed was then increased by steps of 2 m/min every 2 min until the mice was unable or unwilling to carry on despite mild stimulation with a wooden cane. Mice in the HIIT group performed an uphill performance test based on a similar protocol on a treadmill set on a 20° incline. Performance tests were carried out at the beginning and after 5 weeks of training in order to re-adjust training intensity.

### Exercise training

Chronic exercise protocols were adapted from previous works^35–37^, with the following modifications: mice in the MICT group ran for 80 min at an intensity of 50–60% (0% slope) of the maximum speed reached during the last exercise performance test. Mice in the HIIT group performed a high intensity interval uphill training which consisted of 13 series alternating between 4 minutes at an intensity corresponding to 85–90% (20° slope) of the maximum speed reached during the last uphill performance test and 2 minutes of rest. To allow for animal acclimatization, exercise intensity was gradually increased over the first 3 weeks at level (0°) or 20° incline in order to reach the objectives described above. Mice assigned to both exercise groups were exposed to 10 weeks of training consisting of treadmill running 5 days per week.

### Body composition

Lean mass and fat mass were assessed with an EchoMRI 3-in-1 instrument (Echo Medical Systems, Houston, TX) at the beginning and at the end of the training protocol.

### *In vivo* Oral Starch Tolerance Test (OSTT) and Insulin Tolerance Test (ITT)

OSTT and ITT were performed *in vivo* at the end of the 10-week training period. Blood glucose was monitored in overnight fasted mice for two hours following an intraperitoneal injection of human insulin (ITT) or an oral gavage of corn starch solution (OSTT). Blood glucose was measured using Accu-Chek Performa (Roche, USA). For ITT, Neutral Protamine Hagedorn Insulin (human NPH insulin) was diluted down to 0.2 U/ml and administered intraperitoneally (2 U/kg BW). For OSTT, the mice were submitted to an oral gavage of 3 g/kg BW of corn starch solution. Blood glucose was measured 30 min, 60 min, 90 min and 120 min after the intervention.

### Whole blood and serum parameters

Whole blood was harvested right after the death of the mice and a small portion was collected in an EDTA-treated tube and put on ice until analysis. The rest of the blood was collected in a dry tube and allowed to clot at room temperature for 45 min before being centrifuged for 10 min at 2000 g. Serum was collected and frozen at −80 °C until analysis. Serum insulin level was estimated using mouse ultrasensitive insulin ELISA kit provided by Alpco (Alpco Diagnostics, USA). Whole blood HbA1c level was assessed by enzymatic assay kit provided by Chrystal Chem (USA).

### Muscle incubation

Intact *Soleus* muscles from both legs were dissected and incubated for 30 min in Krebs–Henselheit bicarbonate buffer (NaCl 120 mM, KCl 4.8 mM, NaHCO_3_ 25 mM, KH_2_PO_4_ 1.2 mM and MgSO_4_ 1.2 mM, pH 7.4), supplemented with 5 mM Hepes, 5 mM glucose and 0.1% BSA^64^. Gas phase in the medium was maintained at 95% O_2_–5% CO_2_. After 30 min of pre-incubation, muscles were transferred into a fresh medium of identical composition in absence or presence of 12 nM insulin and further incubated for 30 min. Samples were then immediately frozen into liquid nitrogen and stored at −80 °C.

### Mitochondrial enzymes activities

25 mg of frozen tissue was extracted with a potter in 20 volumes of buffer (KCl 175 mM, EDTA 2 mM, and Triton 100 × 0.1%, pH: 7.4), submitted to 3 freeze-thaw cycles and diluted 5 times in the same buffer. Citrate Synthase (CS) and β-Hydroxyacyl-CoA (β-HAD) activities were then determined according to the methods described by Stephenson *et al*.^65^.

#### High resolution respirometry (HRR)

HRR was performed in an Oxygraph-2k (Oroboros, Austria) according to the recommendations of previous authors^66^. Briefly, a small amount of *gastrocnemius* muscle was excised and mechanically separated over ice for a standardised period of 4 min before being chemically permeabilised for 30 min in ice-cold BIOPS added with 50 µg/ml saponin. Tissues were washed with respiration medium (MiR05) before 1 to 3 mg of wet tissue was transferred in a chamber containing 2 ml MiR05 + 20 mM creatine monohydrate. Individual chambers were oxygenated to 450 nmol.ml^−1^ with pure O_2_ and calibrated before muscles fibres were added.

#### Substrate uncoupler inhibitor titration (SUIT) protocol

Palmitoyl-carnitine (0.02 mM) and malate (2 mM) were added to the chambers and the oxygen signal was allowed to stabilise for 5 to 10 min. *Gastrocnemius* fibres were added and leak state was recorded. ADP (2.5 mM) along with MgCl_2_ (1.5 mM) was then added into the chambers for fatty-acid stimulated OXPHOS measurement. The rest of SUIT protocol consisted of the sequential recording of plateau O_2_ consumption for complex I (addition of pyruvate 5 mM), complex I + II (addition of succinate 10 mM), complex II (addition of 0.5 µM rotenone), uncoupled complex II (addition of steps of 0.05 µM Carbonyl cyanide m-chloro phenyl hydrazine (CCCP) until maximal O_2_ consumption is reached). Residual oxygen consumption (ROX) was then evaluated after the addition of 2.5 µM antimycin. Temperature was maintained at +37 °C and the medium was reoxygenated whenever the O_2_ level dropped under 250 µM^66^. Mass-specific O_2_ flux was determined using DatLab V.6 (Oroboros, Autria) from steady-state O_2_ flux normalised to tissue wet weight and adjusted for instrumental background.

### Blotting

50 mg of frozen tissue was added to 20 volumes of NP-40 buffer (Tris HCl 50 mM, NaCl 150 mM, NaF 1 mM, Na_3_VO_4_ 1 mM, Nonidet P-40 1%, Sodium deoxycholate 0.25%, pH: 7.4) supplemented with freshly added protease inhibitor cocktail (P8340, Sigma Aldrich) and phosphatase inhibitor tablets (#88667 Thermo Fisher Scientific, USA). The tissues were homogenised on ice using a glass potter before samples were centrifuged at 14000 g for 10 min (+4 °C) and the supernatant was collected. The protein content of the supernatant was determined using a commercial DC protein assay (Bio-Rad, USA) and all samples were subsequently diluted to a standard concentration and diluted with 2X Laemmli buffer.

Blotting was performed as described by Ennequin *et al*.^67^, with the following specificities: membranes were incubated overnight at 4 °C with primary antibodies against AMPKα (D63G4), p-AMPKα Thr172 (40H9), Akt (pan C67E7), p-Akt thr308 (C31E5E), p-Akt ser473 (D9E), all at concentration of 1:1000, purchased from Cell Signaling (USA), Glut4 (IF8) at concentration of 1/200 purchased from Santa Cruz Biotechnology (USA), MFN-2 (NIAR164) at concentration of 1:2000 and OXPHOS Electron Transfer Chain proteins (ETC) at concentration of 1:1000 purchased from Abcam (USA). After incubation, the membranes were washed with TTBS and exposed to appropriate dilutions of anti-species horseradish peroxidase-conjugated secondary antibodies for 1 h at room temperature. Membranes were then washed 3 times in TTBS before being exposed to an enhanced chemiluminescent solution (Clarity Western ECL; Bio-Rad, USA) for 1 min. Membranes where exposed using a Bio-Rad ChemiDoc system, and band densities were determined using image-analysis software (Image Lab V5.0, Bio-Rad, USA). Stain Free® (Bio-Rad, USA) blot image was used as total protein loading control and data was normalised accordingly^68^.

### Quantitative Real-Time PCR

Total RNA was extracted from *gastrocnemius* tissue using TRIzol® (Invitrogen, Life Technologies). cDNA was synthesised from 2 μg RNA with the High Capacity cDNA transcription kit (Applied Biosystems, Life Technologies). PCR amplification was carried out using the CFX Bio-Rad system with Taqman probes sets (Applied Biosystems, Massachusetts, USA) and the ΔΔCt method was used to quantify mRNA levels. Gene expression was normalised using Gapdh as a housekeeping gene. Data are represented using the Rq which is normalised to the control group or Rq = 2 − ΔΔCt [ΔCt = Ct (target)-Ct (Gapdh); ΔΔCt = ΔCt (sample)-ΔCt (control)].

### Statistical analysis

Results are shown as the mean ± SEM. Normality was verified using Kolmogorov-Smirnov’s test. Homogeneity of variance was assessed using Bartlett F test. When both conditions of normality and homogeneity of variance were respected, one-way, two-way, three-way or repeated measures ANOVA were run and a Student-Newman-Keuls post-hoc test was applied when ANOVA reached significance level (p < 0.05). When normality was not respected, a Krustal-Wallis test was run instead of ANOVA. When homogeneity of variance was not respected, ANOVA was run with the Greenhouse-Geisser correction.

## Electronic supplementary material

Supplementary figures
