# Supplementary material for: Effects of high-intensity interval training and moderate-intensity continuous training on glycaemic control and skeletal muscle mitochondrial function in db/db mice
Source: Sci Rep. 2017 Mar 16;7:204. doi: 10.1038/s41598-017-00276-8 (PMC5427962; doi:10.1038/s41598-017-00276-8)
Supplement: Supplementary file 1 — Supplementary figures [file 41598_2017_276_MOESM1_ESM.pdf]

Vivien Chavanelle, Nathalie Boisseau, Yolanda F. Otero, Lydie Combaret, Dominique Dardevet, Christophe Montaurier, Geoffrey Delcros, Sébastien Peltier, Pascal Sirvent

Effects of high-intensity interval training and moderate-intensity continuous training on glycaemic control and skeletal muscle mitochondrial function in db/db mice

Figure S1

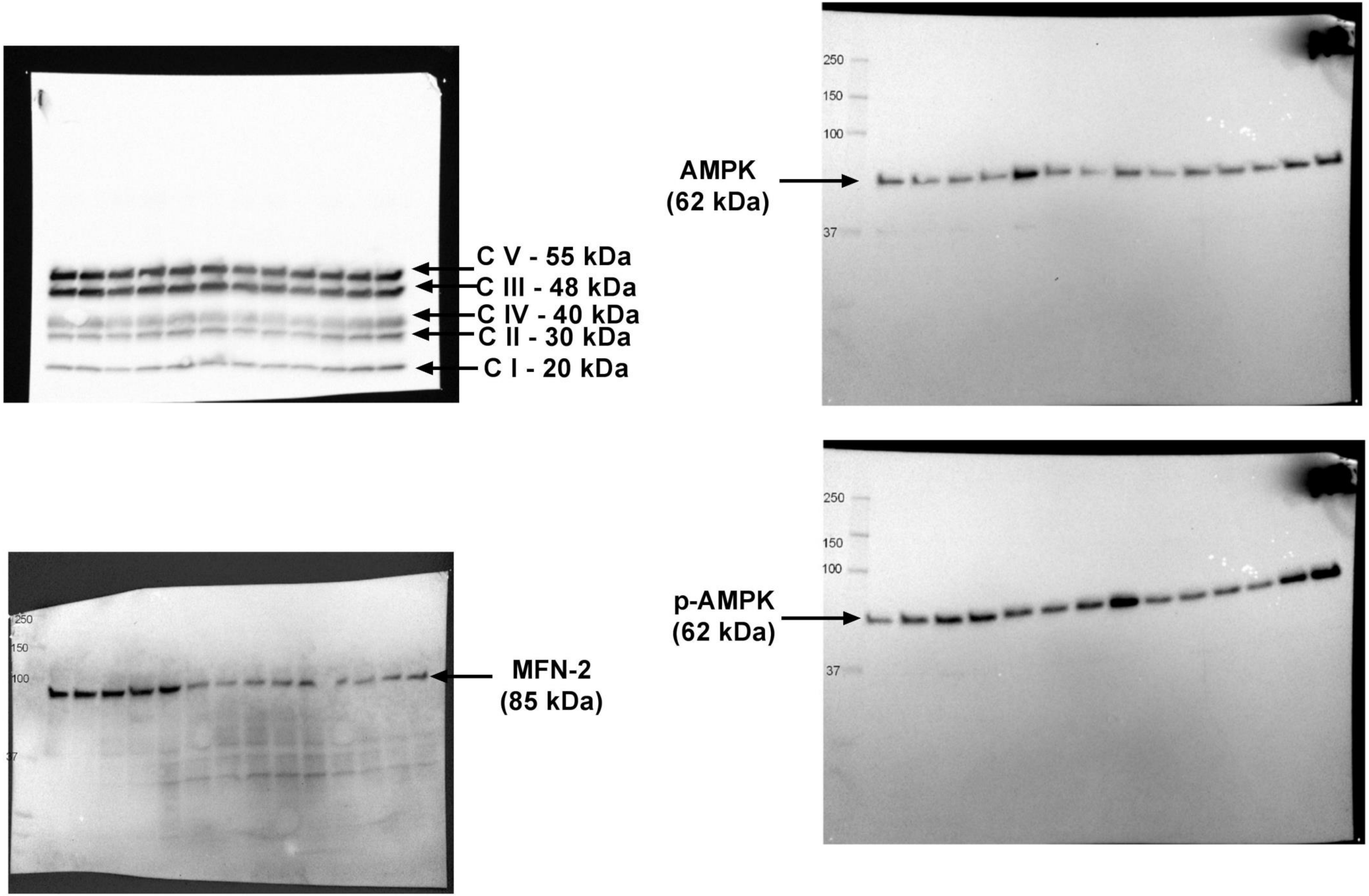
C V - 55 kDa
C III - 48 kDa
C IV - 40 kDa
C II - 30 kDa
C I - 20 kDa
AMPK
(62 kDa)
250
150
100
37
MFN-2
(85 kDa)
250
150
100
37
p-AMPK
(62 kDa)
250
150
100
37

Figure S2

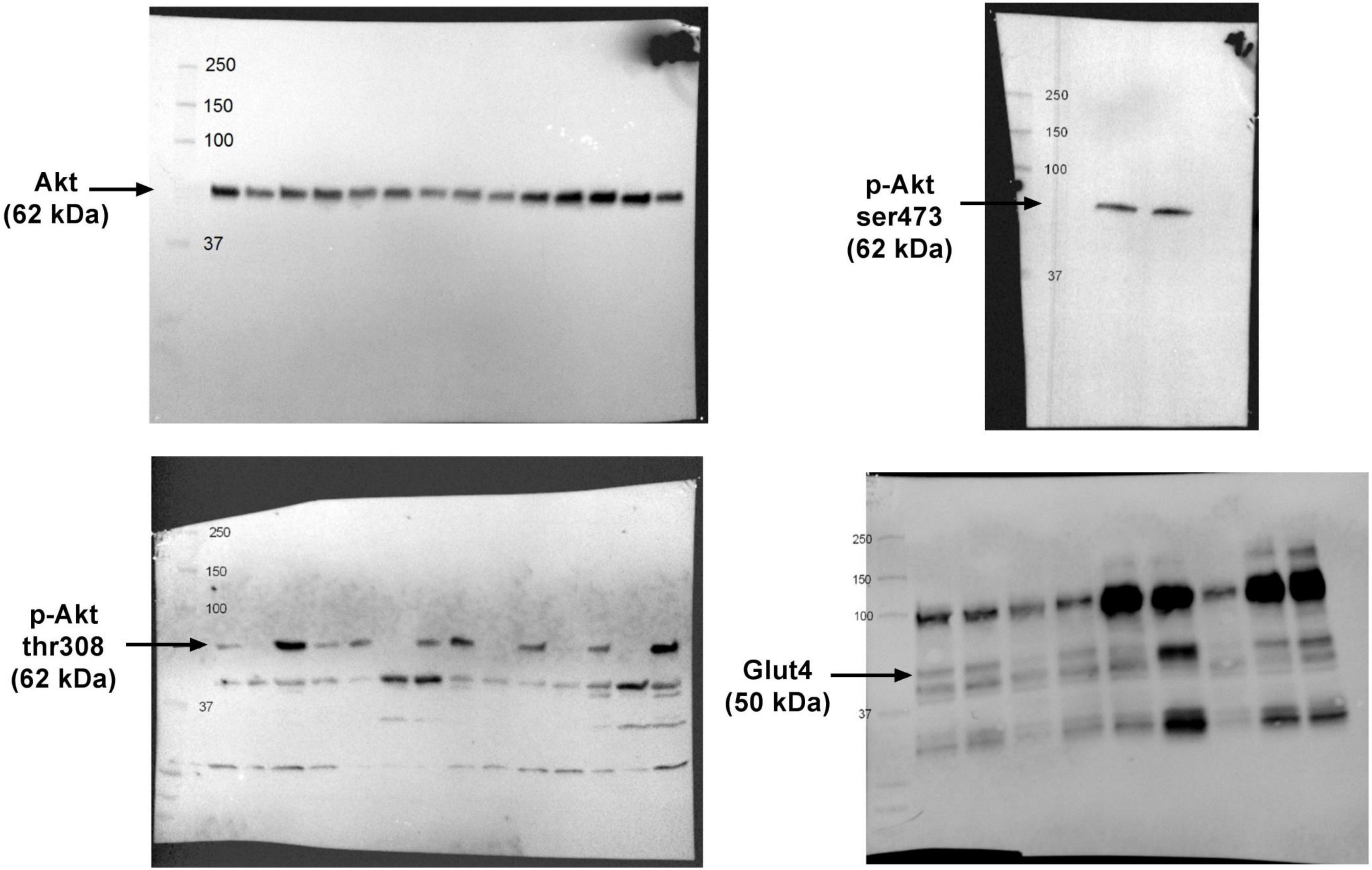
250
150
100
Akt
(62 kDa)
37
250
150
100
p-Akt
ser473
(62 kDa)
37
250
150
100
p-Akt
thr308
(62 kDa)
37
250
150
100
Glut4
(50 kDa)
37

Supplementary figures legend

- Supplementary figure S1: Full scans of panel from Figure 2. Chemiluminescent signals were detected and recorded by exposure of the membranes in a Bio-Rad ChemiDoc system, and band densities were determined using image-analysis software (Image Lab V5.0, Bio-Rad, USA).
- Supplementary figure S2: Full scans of panel from Figure 4. Chemiluminescent signals were detected and recorded by exposure of the membranes in a Bio-Rad ChemiDoc system, and band densities were determined using image-analysis software (Image Lab V5.0, Bio-Rad, USA).
